# Association of information technology ability, workplace social engagement, and successful ageing: validation of a short measure with three African samples

**DOI:** 10.1038/s41598-024-69133-9

**Published:** 2024-08-13

**Authors:** Nestor Asiamah, Sylvester Hatsu, Faith Muhonja, Confidence Chinwe Opara, Frank Frimpong Opuni, Emelia Danquah, Sarra Sghaier

**Affiliations:** 1 Division of Interdisciplinary Research and Practice, School of Health and Social Care, University of Essex, Colchester, Essex CO4 3SQ UK; 2 Department of Geriatrics and Gerontology, Africa Center for Epidemiology, P. O. Box AN 18462, Accra, Ghana; 3 Department of Computer Science, Accra Technical University, Barnes Road, Accra Metro, P. O Box GP 561, Accra, Ghana; 4 Department of Community Health, School of Public Health, Amref International University, P. O. Box 27691, Nairobi, 00506 Kenya; 5 Department of Banking and Finance, College of Management Sciences, Michael Okpara University of Agriculture Umudike, Umuahia, Abia State Nigeria; 6 Department of Marketing, School of Business, Accra Technical University, Barnes Road, Accra Metro, P. O Box GP 561, Accra, Ghana; 7 Research Directorate, Koforidua Technical University, Eastern Region, Post Office Box KF-981, Koforidua, Ghana

**Keywords:** Diseases, Health occupations

## Abstract

This study examined the association of workplace social engagement (WSE) and information technology ability (ITA) with successful ageing and validated a brief scale measuring WSE. The interaction of WSE and ITA on successful ageing was also assessed. A cross-sectional design was adopted, and the participants were 1186 older adults living in Kenya (n = 350), Nigeria (n = 260), and Ghana (n = 576). Pearson’s correlation and factor analyses of two datasets (i.e., waves 1 and 2) from the sample were utilised to validate the WSE scale. Hierarchical linear regression analyses with relevant sensitivity analyses were utilised to assess the associations with wave 2 data. The WSE scale produced satisfactory psychometric properties (i.e., reliability and validity) as a unidimensional measure. WSE and ITA were positively associated with successful ageing in Kenya and Ghana and in the consolidated data. The interaction between WSE and ITA was positively associated with successful ageing and its domains (i.e., illness avoidance, functioning, and engagement with life) in Kenya, Ghana, and consolidated data. At higher ITA or the use of information technologies, WSE is less strongly associated with successful ageing. WSE is more strongly associated with successful ageing only at moderate ITA.

## Introduction

Research has shown that social engagement, also referred to as social activity, can be associated with health^1–4^. Specifically, a systematic review^1^ found a positive association between social engagement and health-related quality of life. According to a prospective study undertaken in Germany, social engagement can buffer depressive symptoms and improve quality of life^3^. A cross-sectional study in South Korea^2^ confirms that social engagement is positively associated with self-reported health.

Social engagement refers to participation in social activities that offer opportunities for social inclusion and volunteering in the community or at work^5^. Researchers recognise social engagement as a normative life behaviour by which life is enjoyed, services are used, and health is maintained^5–7^, which implies that socially disengaged individuals may have lower life satisfaction and are more susceptible to disease and untimely mortality. Hence, social engagement can be associated with successful ageing, which is the maintenance of functional ability necessary for well-being in later life^8^. Rowe and Khan^9^ conceptualised successful ageing as an indicator of ageing in optimal health. Successful ageing is not necessarily about the absence of a disease but concerns the ability to remain engaged with life through employment as well as social and economic activities^10^. It is necessary for social and economic independence in later life.

Employment is an opportunity for people to achieve successful ageing through social engagement. Full-time employees can spend up to 8 h of their weekday time at work^11^, which is long enough to provide opportunities for workplace social engagement. All employees go through the ageing process, and an important public health goal is to enable them to retain gainful employment beyond the retirement age. Maintaining Workplace Social Engagement (WSE) is a potential way to achieve the afore-mentioned goal and maintain health. We define WSE as the individual’s social support for others and their participation in group and volunteering activities at work. This definition was informed by previous studies^5,12^ recognising volunteering, social support (i.e., support for peers at work), and group activities as employee behaviours suited for productivity. These three behaviours can be consistently performed by employees at work while performing their job duties. An existing tool measuring social engagement in the community^13^ incorporates items that could be adapted to measure these behaviours. This tool may be more suited for older employees because it is relatively brief and would not be an excessive burden to the limited vision and functional ability of older people.

The rapid ageing of the workforce requires a tool focused on WSE. It is estimated that the population of older adults in the world would more than double by 2050^14,15^, and a large proportion of older adults are expected to live in Africa by this date. This situation implies that the workforce will be older by 2050. Since individuals lose functional ability as they age^16^, morbidity in the workforce would be high in the future. If so, early retirement of staff and absences from work due to ill-health may overwhelm organizations and weaken their agility. Workplace interventions fostering successful ageing among employees are, thus, necessary. An example of such interventions is rolling out corporate policies to enhance WSE.

As the pieces of evidence provided earlier suggest, WSE can be an important determinant of successful ageing, which is about illness avoidance and the maintenance of physical functioning and engagement with life in old age^10^. In response to global population ageing, studies^17,18^ have recently called for programmes aimed at employees’ successful ageing. Although previous studies have reported a positive association between social engagement and health indicators as reported above, no study has examined the association of WSE with successful ageing among employees, although this relationship would better inform workplace healthy ageing programmes.

Research suggests that Information Technology Ability (ITA) facilitates social engagement through the use of the internet and social media^19^. ITA is not only about the ability to use information technologies but also involves the actual use of these technologies^20^. We argue that ITA may predict WSE for two reasons. Firstly, individuals are more likely to acquire and apply ITA at work since organizations often embark on professional development programmes that improve the skills of their employees. ITA acquired at work can facilitate social engagement within and outside the organization since this skill enables individuals to organize social and recreational events using software such as outlook emails, WhatsApp, and Telegram. Often, employees in an organization arrange lunch time activities with peers through emails and social media. The use of emails, WhatsApp and other social media applications are part of ITA^19^. Further to this, organizations provide access to information technologies that may not be available to the employee at home. Conversely, high ITA may accompany excessive use of information technologies among employees, resulting in social isolation, loneliness, and poor health^21^. From this viewpoint, WSE would be less positively associated with successful ageing at high ITA.

Deductively, ITA may predict WSE and modify the relationship between WSE and successful ageing. An interaction of WSE and ITA on successful ageing is more likely in workplaces where training and other supports are available, and this interaction is worth assessing because it can provide lessons for practice. Because employees spend much of their time at work, their WSE and its influence on health may be significant. Yet, there is a paucity of research on WSE and there is no scale for measuring it. This shortcoming of the literature is worth addressing since three social activities (i.e., volunteering, peer support, and group activity) are traditionally practised in workplaces^22,23^. A scale measuring these activities in the community exists^13^, but this has not been validated in a work context.

Given the above gaps in the literature, this study aimed to validate a WSE scale and assess the association between ITA, WSE, and successful ageing. To meet the above aim, the following research questions are answered*:* (1) what is the factor structure of the WSE engagement scale as assessed with factor analysis, (2) is there an association between WSE and successful ageing, (3) is ITA associated with successful ageing, and (4) does ITA interact with WSE on successful ageing? With the fourth research question, we ascertained whether ITA differentiates the association of WSE with successful ageing. The relationships (regarding research questions 2, 3, and 4) were examined with samples from Kenya, Nigeria, and Ghana to ascertain whether they are consistent across contexts.

This study is significant for some reasons. Firstly, it is the first to validate a tool for measuring WSE, which means it can encourage research on WSE. A brief measure of WSE among older adults is needed because this group may be unable to complete longer questionnaires owing to their physical and cognitive limitations. Researchers^24,25^ insinuate that results from quantitative studies conducted across countries are more useful than results from similar studies based on a sample from a single locality or country. Studies utilising multi-national samples demonstrate consistency of evidence and cultural sensitivity of their results since every country has a unique culture. Scales validated with multi-national samples are also known to be better suited for multiple contexts if they yield satisfactory psychometric properties across the samples^26,27^. Hence, we employed samples from three African countries to answer the above research questions.

## Methods

### Design

**Figure 1:**
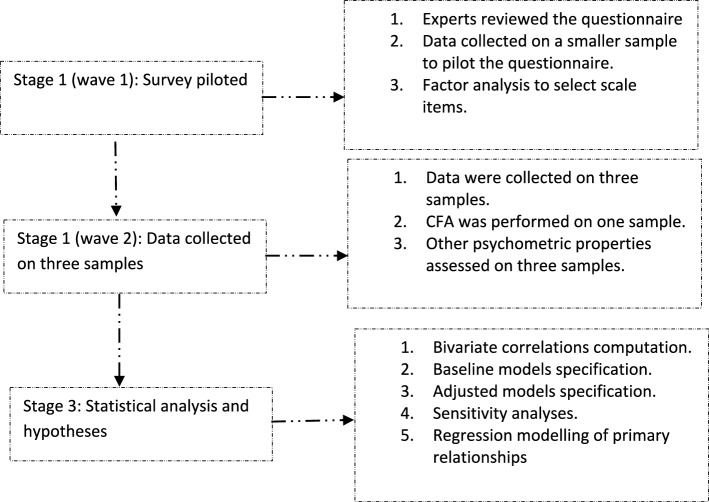
A flow chart of the study design. *CFA* confirmatory factor analysis.

A psychometric test with a cross-sectional design compliant with the CROSS (i.e., Consensus-Based Checklist for Reporting of Survey Studies) checklist^28^ was adopted. Figure 1 is a flowchart describing the study design.

### Participants and selection

The participants were adult employees aged 50+ years in three English-speaking African countries, namely Ghana, Nigeria, and Kenya. These countries were included in this study owing to their availability. The participants were selected with the following four inclusion criteria: (1) being an adult aged 50+ years; (2) having a minimum of basic education, which was an indicator of the ability to complete questionnaires in English; (3) being an employee in any organization, (4) availability for completing surveys at both waves of data collection, and (5) willingness and availability to participate in the study. Some of the researchers supervised the recruitment of participants at corporate institutions, supermarkets, and community centres in Umuahia (Nigeria), Nairobi (Kenya), and Accra (Ghana). A total of 1209 residents from the three cities (Nigeria = 271; Kenya = 363; Ghana = 575) were recruited. We utilized the G*Power tool to calculate the minimum sample necessary for this study. The minimum sample reached with parameters (significance level = 5%; effect size = 0.2, power = 0.8, and a maximum of 11 predictors) recommended in the literature^19^ for a hierarchical linear regression analysis was 95. The power resulting from a sample of 95 (degree of freedom for the denominator—83, and degree of freedom for the numerator—11) was 0.804, which is sufficient for our analysis^29^. A small effect size and a power not less than 0.8 enabled us to reach a minimum sample sufficient for detecting weak effects. There is a consensus in the literature that statistical power and the precision of effect estimates increase as sample size increases^30,31^. Moreover, external validity can be higher with larger samples^30^. For these reasons, we included all 1209 recruited participants in the study.

Following a stepwise approach to the validation of scales^26,32^, two waves of data collection were performed to collect enough data to validate the WSE scale and assess our primary relationships. The use of multiple waves of data allows researchers to demonstrate the consistency of results between samples as well as between exploratory and confirmatory factor analyses^32^. Multiple waves also enabled us to assess the consistency of some psychometric properties between samples or waves. This approach is considered more robust compared to the use of single (isolated) samples. The first wave or sample is often used for understanding the factor structure of a scale through Exploratory Factor Analysis (EFA)^32^. The other wave or sample is used for estimating psychometric properties of the scale resulting from EFA through Confirmatory Factor Analysis (CFA).

Questionnaires were administered in person by some of the researchers and their research assistants. Wave 1 data were collected between 2nd and 10th November 2022 on only the Ghanaian sample. A total of 161 questionnaires in this wave were returned and analysed. Wave 2 data were collected on the three samples between 18th November and 23rd December 2022. Out of 1209 questionnaires sent out for completion in wave 2, 1186 were incorporated into the final analysis. Thus, 23 questionnaires were discarded because they were partially completed. This study received ethical review and clearance from the ethics review board of Africa Centre for Epidemiology (# 004-11-2022-ACE). Ethical review in Nigeria (# 132-22; Date—20th October 2022) and Kenya (# 2313; Date—10th October 2022) was performed by the National Health Research Ethics Committee and National Scientific and Ethics Committee respectively following ethical approval in Ghana. Ethical approval in these countries was waived because ethical clearance was already provided in Ghana, a country with almost the same ethical regulations as Nigeria and Kenya. All ethical considerations, including guidelines from the Helsinki Declaration, were followed. Every participant provided written informed consent after reading details about the study’s potential significance and risks.

### Measurement

Workplace social engagement was operationalised as the frequency of social activities performed by the individual over the past week^13^. It was, therefore, measured by adapting an existing 8-item scale developed to measure the frequency of neighbourhood-level social activities performed^13^. It accompanied 4 descriptive anchors (i.e., not at all—1, rarely—2, sometimes—3, and always—4) describing how often individuals performed social activities over the past 7 days. Examples of items in the scale are “I interacted with colleagues or friends at work” and “I made new friends at work”. This tool was deemed useful for some reasons. First, it constitutes the primary factors recognised as indicators of social engagement in key definitions and frameworks^5,22^. Its domains (i.e., volunteering, organized or group activities, and support for others) could be modified into primary workplace activities that support health and productivity^22^. Finally, the tool is short and suited for older employees with potential physical limitations.

In the original scale, volunteering comprises two items measuring participation in community activities (e.g., cleaning public places) aimed at community welfare. Organised activity comprises three items measuring participation in activities in groups or with others. Support for others contains three items measuring how often one makes new friends and supports them to overcome personal challenges. The scale was adapted by rewording its eight items into activities performed within the workplace. In the adapted scale, volunteering measures social activities beneficial to the organization, not the individual. Organised activity comprises three items measuring participation in activities in groups or with others. Support for others measures how often one makes new friends and supports them. Three researchers (with a PhD and research interests in technologies for ageing) reviewed the original items and determined whether they sufficiently represent or describe social activities in a work context. Appendix A1 shows the original and adapted items. Results of our validation of this scale are presented later in this paper.

We adopted in whole an 18-item scale with five descriptive anchors (i.e., 5—strongly agree, 4—agree, 3—somewhat agree, 2—disagree, and 1—strongly disagree) to measure successful ageing. This tool measures the individual’s situations and experiences over the past seven days. Some items of this scale are “I was healthy enough to move around freely” and “I had enough energy for daily life”. The three domains of this scale are illness avoidance (4 items), engagement with life (i.e., 9 items measuring engagement in social, economic, or work-related activities), and functioning (i.e., 5 items measuring the ability to perform physical tasks unaided). This tool, whose items are shown in Supplementary Appendix A2, produced satisfactory internal consistency in the form of Cronbach’s α ≥ 0.7 with data from wave 2 (i.e., Ghana = 0.85; Kenya = 0.85; Nigeria = 0.88, whole scale = 0.87).

We utilised a 13-item scale and its descriptive anchors (5—strongly agree, 4—agree, 3—somewhat agree, 2—disagree, and 1—strongly disagree) in whole to measure ITA. This tool was adopted in whole from Yu and Chao^20^ and measures how well the individual experimented with new technologies and used them to perform tasks over the past week. Some items of the scale are “In general, I routinely use the Internet to obtain good information” and “I could describe the package software functions”. Supplementary Appendix A3 shows items of the scale, which as a unit produced satisfactory internal consistency across the three samples (i.e., Ghana = 0.8; Kenya = 0.94; Nigeria = 0.88, whole scale = 0.89). We chose the above scales for measuring ITA and successful ageing because their items described experiences in Ghana. The ITA scale was previously used in Ghana^19^ and produced satisfactory psychometric properties.

We measured 11 variables as potential cofounders of the primary association assessed. Self-reported health was measured as a dichotomous variable (poor health—1, and good health—2) and coded into a dummy-type variable in data analysis. Other variables measured as dichotomous variables are gender (male—1, and female—2), job type or schedule (part-time—1, and full-time—2), whether the individual had training on Information Communication Technologies (ICT) at least once in the past year (no ICT training—1, and ICT training—2), and marital status (not married—1, and married—2). Sector was a categorical variable with three groups (manufacturing—1, services—2, and both manufacturing & services—3) that measured the sector (s) in which the individual worked. Five variables (i.e., job tenure, age, physical function, income, and education) were measured as discrete variables. Job tenure was measured by asking the participants to report how long (in years) they had worked in their current organization. Age was the individual’s chronological age whereas physical function was measured following a previous study^19^ by asking the participants to rate on a scale of 1 to 4 (i.e., 1—not at all, 2—low extent, 3—moderate extent, 4—high extent) the extent to which they could perform physical tasks such as walking unaided for at least 10 min in the past week. Income was measured as the individual’s net monthly income reported in United States dollars whereas education was the individual’s years of schooling.

### Common methods bias assessment

We followed Herman’s one-factor method to assess common methods bias by utilising exploratory factor analysis (with varimax rotation) to examine the factor structures of all scales. This analysis, which was based on data from wave 2, yielded a factor structure comprising three factors for ITA and successful ageing and a factor for WSE (with factor loadings ≥ 0.5). As recommended in the literature^33,34^, the first factor for ITA and successful ageing produced a variance < 40%. This result confirmed the absence of common methods bias in the data.

### Statistical analysis

Data were analysed with the SPSS 28 (IBM Inc., New York, USA) and Amos in two stages. We validated the WSE scale with EFA and CFA in the first stage and tested the hypothesised relationships with HLR in the second stage. Before analysing the data at these stages, we performed exploratory analysis to summarise the data and assess key assumptions governing the use of the chosen statistical tools. Discrete and categorical variables were summarised with averages and counts respectively. The proportion of missing data was less than 2%, so we utilised “listwise deletion” to remove all missing data^35^. Five relevant assumptions including linearity of the hypothesized relationships and normal distribution of the data were then performed through SPSS. These assumptions govern the use of HLR, EFA, and CFA. Supplementary Appendix B1 shows the results of these analyses and the procedures followed in them. Following previous research^19^, we performed the first sensitivity analyses to screen for the ultimate confounders, which are covariates likely to confound the hypothesized relationships. This analysis was carried out to ensure that variables unlikely to confound the hypothesised relationships were removed from the analysis. Multi-collinearity in multiple regression analysis increases as the number of predictors increases^36^, so it was necessary to remove unnecessary covariates (predictors) from the analysis. Education and age were retained in the sensitivity analysis as the ultimate confounders and were included in the ultimate models. Supplementary Appendix B2 shows the steps taken in this analysis.

#### Stage 1—scale validation

We first performed an EFA with varimax rotation to assess the scale’s factor structure using data from wave 1. The EFA through SPSS produced a unidimensional factor structure and retained all the original items at factor loading ≥ 0.5. The total variance in this analysis was 58.1%. We then specified a structural model on data from wave 2 through Amos to ascertain if the EFA results were consistent with CFA. The unidimensional factor structure was confirmed with CFA through Amos at satisfactory fit indices in the form of the chi-square = 8.12 (p = 0.775), goodness-of-fit index = 0.99, Tucker-Lewis index = 1.00, and root mean square error of approximation = 0.000^37^. Since a unidimensional factor structure was suggested by both EFA and CFA, we adopted a previous procedure unique to a unidimensional measure^38,39^ to further validate the scale.

In further validating the scale, we utilised SPSS to assess criterion-related validity, internal consistency, inter-rater reliability, and Pearson’s correlation among the 8 items of WSE based on both waves of data^38–40^. A satisfactory minimum correlation coefficient ≥ 0.2 at p < 0.001 was reached^38^. We assessed criterion-related validity by computing the correlation of WSE (which is formed by summing all 8 scale items) with engagement with life (another measure of social engagement from the successful ageing scale) and self-reported health. The correlation between WSE and engagement with life (r = 0.44–0.51, p < 0.001) and self-reported health (r = 0.15–0.27, p < 0.001) was significant. Cronbach’s α (wave 1 = 0.9, and wave 2 = 0.8) was satisfactory^37,41^. Finally, we reached a satisfactory inter-rater reliability (wave 1 = 0.9, and wave 2 = 0.79)^40^. Supplementary Appendix C shows tabulated results of our scale validation. Scores ranged from 1 to 32 on the WSE scale, with larger scores representing higher social engagement.

#### Stage 2—testing the hypothesised relationships

**Figure 2:**
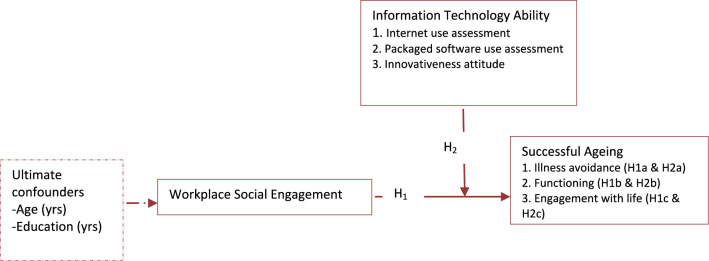
A conceptual model of the association of WSE and ITA with successful ageing. H_1_—workplace social engagement is associated with successful ageing; H_1a_—workplace social engagement is associated with illness avoidance; H_1b_—workplace social engagement is associated with functioning; H_1c_—workplace social engagement is associated with engagement with life; H_2_—WSE and ITA interact on successful ageing; H_2a_—WSE and ITA interact on illness avoidance; H_2b_—WSE and ITA interact with functioning; H_2c_—WSE and ITA interact on engagement with life.

In the hypotheses testing phase, we utilised wave 2 data from the three countries to assess Pearson’s correlation between the main variables and ultimate confounders. We utilised wave 2 data to analyse the relationships because they came from all participants and were collected for testing the relationships based on previous research^26,42^. Two groups of regression models were fitted. The first group assessed the association of WSE, ITA, and the interaction of WSE and ITA (i.e., WSExITA) with successful ageing without adjusting for the ultimate covariates. The second group (i.e., the ultimate models) built upon groups 1 by incorporating the ultimate confounders. The interaction term WSExITA was created using the compute function in SPSS based on a previous study^43^. With charts plotted in SPSS, we visualised the regression weights between WSE and successful ageing at different levels (i.e., low, medium, and high) of ITA. The statistical significance of the results was detected at a minimum of p < 0.05. Figure 2 shows the hypotheses and conceptual model tested.

## Results

### Demographic characteristics and correlations

In Table 1, about 52% (n = 616) of the participants were men whereas about 91% (n = 1074) were married. The average age of the participants was 57 years (Mean = 56.53; SD = 5.94) and the average successful ageing, WSE, and ITA were respectively 65 (Mean = 64.56; SD = 12.11), 22 (Mean = 22.23; SD = 5.00) and 46 (Mean = 45.68; SD = 10.14).

**Table 1:** Descriptive statistics summarising variables included in the study (n = 1186).

Variable	Group	n/mean	%/SD
Categorical variables			
Country/sample	Kenya	350	29.51
	Nigeria	260	21.92
	Ghana	576	48.57
Self-reported Health	Poor	192	16.19
	Good	994	83.81
	Total	1186	100.00
Training in ICT	No ICT training	632	53.29
	ICT trained	554	46.71
Sector	Manufacturing	810	68.30
	Services	232	19.56
	Manufacturing and Service	144	12.14
Job schedule	Part-time	256	21.59
	Full-time	930	78.41
Gender	Male	616	51.94
	Female	570	48.06
Marital status	Not married	112	9.44
	Married	1074	90.56
	Total	1186	100.00
Discrete variables			
Successful ageing	–	64.56	12.11
Illness avoidance	–	13.72	3.62
Functioning	–	17.89	3.87
Engagement with life	–	32.96	6.75
Workplace social engagement	–	22.23	5.00
Information technology ability	–	45.68	10.14
Physical function	–	2.59	0.90
Job tenure (years)	–	12.02	7.86
Income (USD)	–	216.02	190.41
Education (years)	–	16.50	4.18
Age (years)	–	56.53	5.94

*ICT* information communication technology, *SD* standard deviation, *n* frequency, % percent; frequency (n) and % apply to only categorical variables whereas the mean and standard deviation apply to only discrete variables.

In Table 2, there was a strong positive correlation between successful ageing and WSE within the consolidated data (r = 0.513, p < 0.001, two-tailed), which means higher WSE was associated with higher successful ageing. ITA was also positively correlated with WSE across the three samples and in the consolidated data (r = 0.42–0.70, p < 0.001, two-tailed). Thus, older employees with higher ITA reported higher WSE. ITA was positively correlated with successful ageing (r = 0.24–0.70, p < 0.001, two-tailed), which suggests that older adults with higher ITA reported higher successful ageing. All the above correlations were stronger for Kenya and Ghana (Table 3).

**Table 2:** Bivariate correlations between information technology ability, workplace social engagement, and successful ageing.

Variable	1	2	3	4	5	6	7	8
Kenya (n = 350)								
1. Successful ageing	1	0.689**	0.828**	0.891**	0.638**	0.693**	− 0.06	− 0.227**
2. Illness avoidance		1	0.526**	0.372**	0.580**	0.598**	0.003	− 0.109*
3. Functioning			1	0.588**	0.500**	0.577**	− 0.054	− 0.202**
4. Engagement with life				1	0.513**	0.555**	− 0.073	− 0.218**
5. Workplace social engagement					1	0.696**	− 0.038	− 0.226**
6. Information technology ability						1	0.082	− 0.212**
7. Education (years)							1	0.124*
8. Age (years)								1
Nigeria (n = 260)								
1. Successful ageing	1	0.661**	0.852**	0.870**	0.163**	0.237**	0.089	0.024
2. Illness avoidance		1	0.505**	0.283**	0.101	0.106	− 0.017	− 0.135*
3. Functioning			1	0.613**	0.228**	0.230**	0.09	0.055
4. Engagement with life				1	0.094	0.219**	0.113	0.088
5. Workplace social engagement					1	0.423**	− 0.057	0.019
6. Information technology ability						1	0.145*	− 0.031
7. Education (years)							1	0.289**
8. Age (years)								1
Ghana (n = 576)								
1. Successful ageing	1	0.859**	0.871**	0.932**	0.580**	0.546**	− 0.067	− 0.125**
2. Illness avoidance		1	0.680**	0.691**	0.466**	0.461**	− 0.035	− 0.124**
3. Functioning			1	0.699**	0.465**	0.416**	− 0.054	− 0.056
4. Engagement with life				1	0.584**	0.550**	− 0.079	− 0.140**
5. Workplace social engagement					1	0.614**	0.039	− 0.114**
6. Information technology ability						1	0.207**	− 0.052
7. Education (years)							1	0.076
8. Age (years)								1
Consolidated (n = 1186)								
1. Successful ageing	1	0.740**	0.857**	0.905**	0.513**	0.581**	− 0.026	− 0.126**
2. Illness avoidance		1	0.576**	0.461**	0.440**	0.471**	− 0.061*	− 0.129**
3. Functioning			1	0.655**	0.427**	0.486**	0.003	− 0.085**
4. Engagement with life				1	0.439**	0.510**	− 0.016	− 0.108**
5. Workplace social engagement					1	0.613**	− 0.024	− 0.122**
6. Information technology ability						1	0.115**	− 0.101**
7. Education (years)							1	0.155**
8. Age (years)								1

**p < 0.001; *p < 0.05.

**Table 3:** The associations among information technology ability, workplace social engagement, and successful ageing.

Predictor	Kenya (n = 350)		Nigeria (n = 260)		Ghana (n = 576)		Consolidated (n = 1186)	
	β (t)	SE	β (t)	SE	β (t)	SE	β (t)	SE
Non-adjusted models								
(Constant)	(10.63)**	1.991	(12.90)**	13.68	(14.539)**	6.174	(19.671)**	7.282
WSE	0.302 (5.886)**	0.117	0.077 (1.147)	0.667	0.394 (9.56)**	0.289	0.242 (8.211)**	0.358
ITA	0.482 (9.939)**	0.056	0.205 (3.071)*	0.300	0.304 (7.394)**	0.142	0.427 (0.427)**	0.166
WSE × ITA	0.717 (19.21)**	0.001	0.222 (3.662)**	0.002	0.621 (18.957)**	0.001	0.589 (25.095)**	0.001
Adjusted models								
(Constant)	(4.654)**	10.224	(5.824)**	15.043	(11.910)**	6.622	(11.190)**	10.630
WSE	0.272 (5.211)**	0.290	0.085 (1.260)	0.674	0.322 (7.661)**	0.286	0.232 (8.001)**	0.417
ITA	0.495 (9.552)**	0.150	0.192 (2.814)*	0.301	0.349 (8.371)**	0.141	0.438 (14.919)**	0.197
WSE × ITA	0.702 (18.508)**	0.001	0.218 (3.587)**	0.014	0.634 (19.404)**	0.006	0.585 (24.797)**	0.007
Education (years)	− 0.084 (− 2.250)*	0.168	0.063 (0.972)	0.178	− 0.149 (− 4.527)**	0.069	− 0.065 (− 2.748)*	0.138
Age (years)	− 0.048 (− 1.275)	0.128	0.010 (0.163)	0.130	− 0.055 (− 1.687)	0.038	− 0.043 (− 1.842)	0.099

*WSE* workplace social engagement, *ITA* information technology ability, *SE* standard error (of B, the unstandardised coefficient), *β* standardised coefficient (effect size); values in parentheses represent t-statistics from multiple linear regression analysis; the F-test for each model was significant at p < 0.001; the Durbin–Watson statistic ranged between 1.78 and 2.03 for the multiple models; the tolerance values for all predictors in the multiple models ranged between 0.5 and 0.99; the adjusted R-square ranged between 20.1 and 43.2% for the simple models and between 40.4 and 65.3% for the multiple models.

**p < 0.001; *p < 0.05.

### Main results

After adjusting for the ultimate covariates in Table 3, WSE was positively associated with successful ageing in the consolidated data (β = 0.23, SE = 0.42; t = 8.00, p < 0.001) as well as in Kenya (β = 0.27, SE = 0.29; t = 5.21, p < 0.001) and Ghana (β = 0.32, SE = 0.29; t = 7.66, p < 0.001) but not in Nigeria (β = 0.09, SE = 0.67; t = 1.26, p > 0.05). This result confirmed that older employees reported higher successful ageing at higher WSE in Ghana and Kenya. ITA was positively associated with successful ageing in the three samples and in the consolidated data at a minimum of p < 0.05, which implies higher ITA was associated with higher successful ageing. This association was stronger for Kenya and Ghana.

The effect sizes of WSE on successful ageing in Kenya, Nigeria, Ghana, and consolidated data were respectively 0.27, 0.09, 0.32, and 0.23 (see Table 3). These effect sizes were increased by ITA to 0.7 (158% increase), 0.22 (156% increase), 0.63 (97% increase), and 0.59 (152% increase) respectively. Corresponding effect sizes in the non-adjusted models were different.

**Figure 3:**
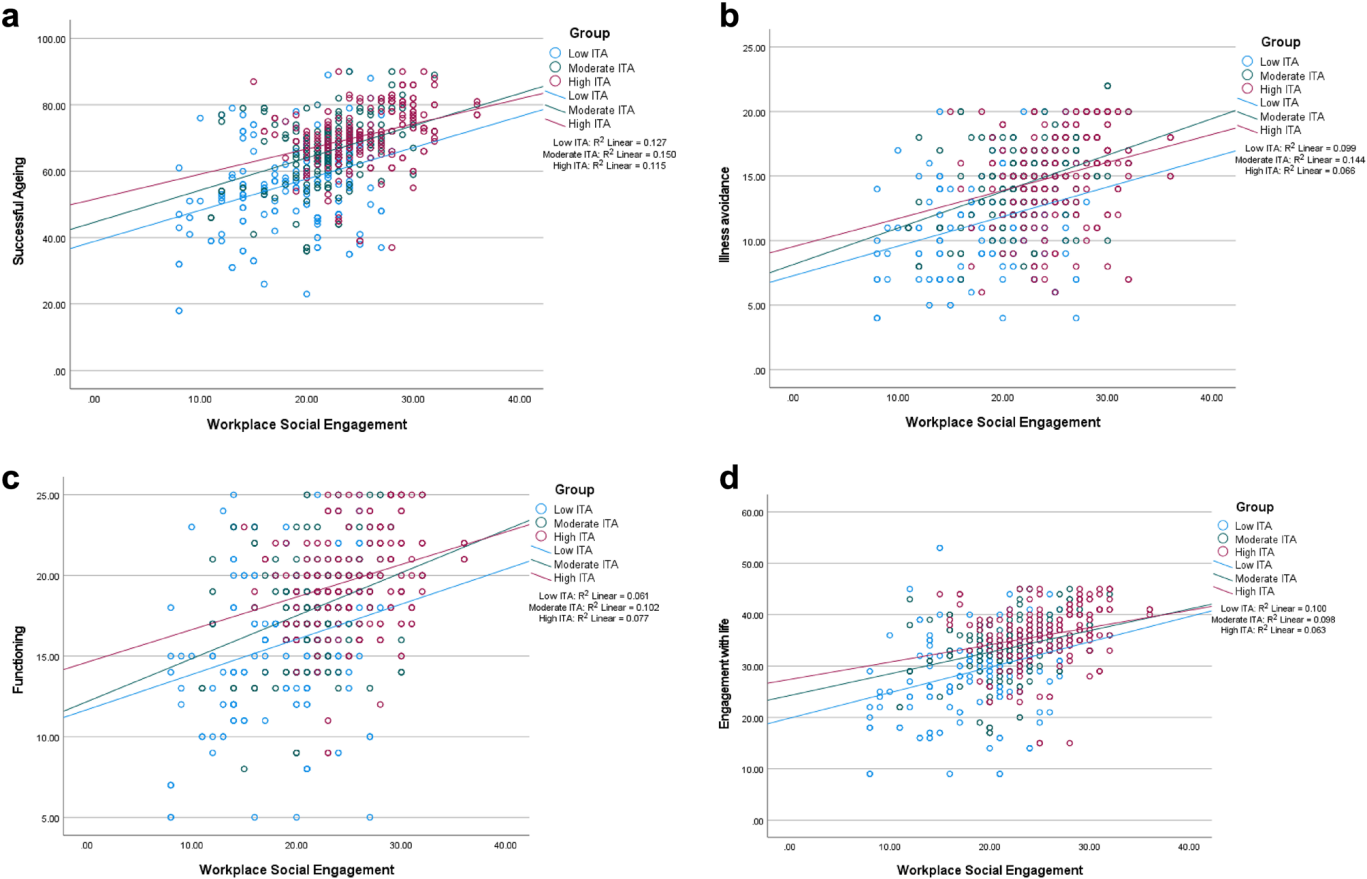
(**a**) The interaction of WSE with ITA on successful ageing (n = 1186). (**b**) The interaction of WSE with ITA on illness avoidance (n = 1186). (**c**) The interaction of WSE with ITA on functioning (n = 1186). (**d**) The interaction of WSE with ITA on engagement with life (n = 1186).

Table 4 shows the association between ITA, WSE, and the three domains of successful ageing. After adjusting for the ultimate covariates in the consolidated data, WSE was positively associated with all domains of successful ageing, where the strongest effect of WSE was on illness avoidance (β = 0.23, SE = 0.14; t = 7.07, p < 0.001). ITA was also positively associated with all domains of successful ageing in the consolidated data, where its strongest effect was on engagement with life (β = 0.4; SE = 0.12; t = 12.56, p < 0.001). The effect sizes of WSE on illness avoidance, functioning, and engagement with life were respectively 0.23, 0.2, and 0.19 in the consolidated data. These were increased by 0.26 (116% increase), 0.30 (148% increase), and 0.31 (164% increase) by ITA respectively. In the three countries, WSE was positively associated with successful ageing at different levels of ITA (see Fig. 3a–d).

**Table 4:** Association of information technology ability, workplace social engagement, and dimensions of successful ageing.

Model	Predictor	Illness avoidance		Functioning		Engagement with life	
		β (t)	SE	β (t)	SE	β (t)	SE
Kenya							
1	(Constant)	(3.665)**	1.872	(8.649)**	2.121	(8.786)**	3.907
	WSE	0.318 (5.532)**	0.095	0.191 (3.179)*	0.107	0.247 (4.058)**	0.197
	ITA	0.377 (6.562)**	0.048	0.444 (7.371)**	0.055	0.383 (6.305)**	0.100
	WSE × ITA	0.624 (14.907)**	0.002	0.586 (13.508)**	0.002	0.578 (13.205)**	0.004
2	(Constant)	(0.035)	3.443	(4.016)**	3.882	(4.467)**	7.127
	WSE	0.321 (5.507)**	0.098	0.168 (2.771)*	0.11	0.219 (3.586)**	0.202
	ITA	0.387 (6.619)**	0.051	0.454 (7.456)**	0.057	0.394 (6.429)**	0.105
	WSE × ITA	0.628 (14.640)**	0.002	0.570 (12.898)**	0.002	0.558 (12.575)**	0.004
	Education (years)	− 0.023 (− 0.545)	0.057	− 0.078 (− 1.772)	0.064	− 0.088 (− 1.997)*	0.117
	Age (years)	0.058 (1.353)	0.043	− 0.069 (− 1.521)	0.049	− 0.081 (− 1.761)	0.089
Nigeria							
1	(Constant)	(7.965)**	4.635	(9.829)**	4.567	(12.069)**	7.797
	WSE	0.068 (0.991)	0.226	0.159 (2.404)*	0.223	0.001 (0.020)	0.38
	ITA	0.077 (1.134)	0.102	0.163 (2.455)*	0.1	0.219 (3.256)**	0.171
	WSE × ITA	0.128 (2.080)*	0.005	0.262 (4.362)**	0.005	0.161 (2.624)*	0.008
2	(Constant)	(5.795)**	5.065	(3.914)**	5.015	(4.505)**	8.543
	WSE	0.076 (1.103)	0.227	0.168 (2.499)*	0.225	0.007 (0.104)	0.383
	ITA	0.067 (0.964)	0.101	0.151 (2.220)*	0.1	0.210 (3.062)*	0.171
	WSE × ITA	0.125 (2.032)*	0.005	0.259 (4.308)**	0.005	0.158 (2.574)*	0.008
	Education (years)	0.018 (0.277)	0.06	0.067 (1.047)	0.059	0.060 (0.929)	0.101
	Age (years)	− 0.140 (− 2.157)*	0.044	0.037 (0.582)	0.043	0.077 (1.213)	0.074
Ghana							
1	(Constant)	(8.499)**	1.984	(12.695)**	2.183	(14.456)**	3.117
	WSE	0.294 (6.495)**	0.093	0.336 (7.307)**	0.102	0.396 (9.661)**	0.146
	ITA	0.280 (6.188)**	0.046	0.209 (4.548)**	0.05	0.307 (7.504)**	0.072
	WSE × ITA	0.507 (14.080)**	0.002	0.497 (13.719)**	0.002	0.367 (9.058)**	0.003
2	(Constant)	(7.650)**	2.149	(8.999)**	2.37	0.355 (8.614)**	3.323
	WSE	0.268 (5.867)**	0.093	0.320 (6.905)**	0.102	− 0.161 (− 4.937)**	0.143
	ITA	0.315 (6.790)**	0.046	0.242 (5.131)**	0.05	− 0.069 (− 2.158)*	0.07
	WSE × ITA	0.511 (14.083)**	0.002	0.511 (13.979)**	0.002	0.635 (19.554)**	0.003
	Education (years)	− 0.105 (− 2.855)*	0.022	− 0.117 (− 3.124)**	0.025	(44.226)**	0.035
	Age (years)	− 0.069 (− 1.913)	0.012	0.002 (0.047)	0.014	0.620 (18.944)**	0.019
Consolidated (n = 1186)							
1	(Constant)	(9.910)**	2.830	(16.417)**	2.957	(17.979)**	4.940
	WSE	0.243 (7.662)**	0.138	0.207 (6.547)**	0.144	0.202 (6.496)**	0.241
	ITA	0.322 (10.171)**	0.065	0.359 (11.350)**	0.068	0.387 (12.433)**	0.114
	WSE × ITA	0.490 (19.346)**	0.003	0.498 (19.755)**	0.003	0.508 (20.319)**	0.005
2	(Constant)	(7.382)**	3.552	(8.258)**	3.756	(9.871)**	6.331
	WSE	0.225 (7.073)**	0.139	0.200 (6.283)**	0.146	0.191 (6.091)**	0.243
	ITA	0.338 (10.573)**	0.066	0.365 (11.357)**	0.069	0.396 (12.560)**	0.115
	WSE × ITA	0.486 (19.151)**	0.003	0.496 (19.494)**	0.003	0.505 (20.028)**	0.005
	Education (years)	− 0.086 (− 3.366)**	0.046	− 0.032 (− 1.233)	0.049	− 0.051 (− 2.044)*	0.084
	Age (years)	− 0.055 (− 2.147)*	0.033	− 0.019 (− 0.749)	0.035	− 0.037 (− 1.475)	0.061

*WSE* Workplace social engagement, *ITA* information technology ability, *β* standardised coefficient (effect size); values in parentheses represent t-statistics from multiple linear regression analysis; the F-test for each model was significant at p < 0.05; the Durbin–Watson statistic ranged between 1.81 and 2.01 for the multiple models; the tolerance values for all predictors in the multiple models ranged between 0.6 and 0.99; the adjusted R-square ranged between 0.07 and 42.3%.

**p < 0.001; *p < 0.05.

As shown in Fig. 3a, WSE was more strongly associated with successful ageing at moderate ITA, compared to low ITA. On the other hand, WSE was less strongly associated with successful ageing at high ITA, compared to low ITA. WSE was less strongly associated with successful ageing at high ITA, compared to moderate ITA. Similar results can be seen in Fig. 3b and Fig. 3c. In Fig. 3d, WSE was less strongly associated with life engagement at moderate and high ITA, compared to low ITA.

## Discussion

This study validated a short scale for measuring WSE and ascertained whether WSE and ITA interact to influence successful ageing. Following our EFA and a previous procedure for validating unidimensional scales^38^, we found that the WSE scale produced satisfactory psychometric properties, suggesting that it could be used as a subjective measure of the frequency of workplace social engagement. Previous studies^38,39^ utilising our approach have produced similar results in the form of factor loadings ≥ 0.5, Cronbach’s α ≥ 0.7, criterion-related validity (i.e., a correlation between the scale and measures of social engagement and health), and inter-rater reliability. Unlike the original scale, which had three domains, this scale is unidimensional, although all the original items were retained. Thus, the three previous dimensions were consolidated into a single questionnaire without domains or factors representing sub-scales. The one-dimensionality of the scale could be due to our rephrasing of the original items in a workplace context. The usefulness of the scale is signified by the consistency of the resulting psychometric properties across African samples.

This study confirmed a positive association of WSE with successful ageing in the consolidated data and in two of the samples (i.e., Ghana and Kenya), which suggests that employees with higher WSE reported higher successful ageing proxied with illness avoidance, functioning, and engagement with life. Thus, employees who reported higher WSE were more likely to avoid illnesses, engage with life, and maintain physical function. Since the three dimensions of successful ageing are indicators of health, this result is analogous to results from studies reporting a positive association between social engagement and health^1–4^. Our study is, nevertheless, unique for being the first study to confirm a link between social engagement and successful ageing. It is also the first to assess and confirm this relationship in a work context. It is worth noting that Nigeria produced the weakest association between WSE and successful ageing, which suggests that the strength of this association may differ significantly across contexts.

ITA was found to be positively associated with successful ageing in the three samples and consolidated data, but the associations were stronger in Kenya and Ghana. ITA had a positive influence on each domain of successful ageing. Although this study is the first to confirm these association, the above results are comparable to results from some previous studies. In the United States, for example, a positive correlation between the ability to use information technologies and health and functioning (which are domains of successful ageing) has been confirmed^44^. Our evidence is also corroborated by several researchers^19,45,46^ who reason that ITA can facilitate health-seeking behaviours. Some of these behaviours are exercise maintenance or adherence, health information seeking, and social participation, which are all likely to occur at work and enhance health^11,22^.

The interaction of WSE and ITA was positively associated with successful ageing and its three indicators (i.e., illness avoidance, functioning, and engagement with life), which suggests that WSE was associated with successful ageing at different levels of ITA. Specifically, WSE was more strongly associated with successful ageing as well as two of its domains (i.e., functioning and illness avoidance) at moderate ITA, compared to low ITA. However, WSE was less strongly associated with life engagement at high ITA, compared to low and moderate ITA. ITA may weaken the association of WSE and successful ageing in a situation where older adults with high ITA do not use their information technology skills in ways that contribute to functioning, life engagement, and illness avoidance. Such older employees may use their skills in excessively chatting with others on phone and social media, which increases the risk of social isolation, loneliness, and other mental health problems^21,47,48^. If these older employees use their ITA in harmful ways persistently, their WSE would be less positively associated with successful ageing. It is possible that high ITA encourages the use of information technologies in a less circumspect or unsafe way, which can weaken the association of WSE with successful ageing. Further to the above, moderate use of information technologies strengthens the positive association of WSE with successful ageing and two of its domains (i.e., illness avoidance and functioning), given that ITA is an indicator of information technology use. Higher levels of use of information technologies, on the other hand, weakens the WSE-successful-ageing nexus.

Our results regarding the association of WSE with successful ageing are supported by the Activity Theory of Ageing (ATA), which makes two dominant assumptions^49,50^. First, people can maintain social engagement and physical activity in later life by adapting previous experiences. Secondly, social engagement enables the individual to maintain health in later life. These arguments suggest that social engagement in later life or among older employees would be associated with health outcomes such as illness avoidance. Our confirmation of the positive association between ITA and successful ageing is also congruent with the ATA’s argument that ageing people can maintain abilities by adapting previous experiences, which include social support from social network members. Although older adults are generally reluctant to use information technologies^45^, those working with organizations are likely to acquire and maintain support for using these technologies. Training and other human development programs (e.g., mentoring, and peer observation) to which older employees are exposed at the workplace may enhance ITA and encourage technology use. Our evidence suggests that behaviours and abilities recognised by the ATA (e.g., social engagement, and the ability to use health-supporting resources such as information technologies) can interact to influence successful ageing.

Although training was not considered a predictor of ITA and successful ageing in this study, our result suggests that investing in any training intended to enhance ITA can increase employees’ social inclusiveness and successful ageing at work. This viewpoint is supported by studies^51,52^ that recognise the role of training in improving ITA. To advance this evidence, future researchers may incorporate training into our analysis to ascertain whether the influence of ITA on WSE and successful ageing depends on it. Depending on outcomes from this or related analysis, organizations may enhance their commitment to successful ageing at work by supporting employees to increase their ITA, specifically through training. Investment in programmes for improving ITA can be rewarding to organizations since illness avoidance can maximise productivity. Organizations can avoid the cost of staff absences due to ill-health if they facilitate successful ageing by enhancing ITA. Thus, continuous ITA enhancement may be a good way to manage employees’ ageing. Yet, organizations ought to ensure older employees do not use information technologies excessively or in ways that increase social isolation and loneliness, given that high ITA may weaken the association of WSE with successful ageing.

### Strengths and limitations

This study has some limitations. We could not assess the test–retest reliability of the scale, which means we could not demonstrate the stability of the scale over time. The cross-sectional design utilised does not establish causation between the variables, although this study provides statistics (e.g., effect sizes) that may guide the design of future studies intended to mitigate this shortcoming. Our findings may have limited generalisability since we used a non-probability sampling method. We, therefore, call for studies utilising representative samples. We used subjective measures, which means our study was prone to response bias. Future researchers are encouraged to use objective measures if possible. The adapted scale may not include all aspects of WSE for every context, so we call for future studies aimed at incorporating more relevant domains into this scale.

This study has several strengths despite the above limitations. Firstly, it sets the foundation for more research for being the first to validate a scale measuring WSE. This study examines the associations with multiple samples rather than a single sample and, thus, demonstrates whether our results are stable between samples from different countries. Our sensitivity analyses maximised the robustness of our design and enabled us to demonstrate the potential influence of confounders on the effect sizes. As the analysis reveals, the regression weights in the ultimate models are different from those in the non-adjusted models. Thus, without the sensitivity analyses, we would have overestimated or underestimated the effect sizes. Finally, this study was compliant with the CROSS checklist, which means it met relevant quality markers^28^.

## Conclusion

The adapted WSE scale produced satisfactory psychometric properties and can, therefore, be used as a unidimensional measure of the frequency of WSE. Successful ageing was positively associated with WSE and ITA, suggesting that older employees with higher ITA and WSE reported higher successful ageing. The interaction of WSE and ITA was positively associated with successful ageing and its three domains (i.e., illness avoidance, functioning, and engagement with life) in all samples, which implies that WSE is positively associated with successful ageing at different levels of ITA. WSE is more strongly associated with successful ageing and two of its domains (i.e., functioning and illness avoidance) at moderate ITA, compared to low ITA. The associations confirmed were stronger in Kenya and Ghana. At moderate ITA or use of information technologies, WSE is more strongly associated with successful ageing. Organizations are encouraged to support their older employees to use information technologies moderately.

## Supplementary Information

Supplementary Information 1.

Supplementary Information 2.

Supplementary Information 3.

## Data Availability

The data used for this manuscript will be made available by the corresponding author upon request.

## References

[1] Tobin, M. C., Drager, K. D. R. & Richardson, L. F. A systematic review of social participation for adults with autism spectrum disorders: Support, social functioning, and quality of life. *Res. Autism Spectr. Disord.* **8**, 214–229 (2014).

[2] Zhao, B., Kim, J. E., Moon, J. & Nam, E. W. Social engagement and subjective health among older adults in South Korea: Evidence from the Korean Longitudinal Study of Aging (2006–2018). *SSM Popul. Health* **21**, 101341 (2023). 36845671 10.1016/j.ssmph.2023.101341 PMC9950723

[3] Hajek, A. *et al.* The impact of social engagement on health-related quality of life and depressive symptoms in old age—Evidence from a multicenter prospective cohort study in Germany. *Health Qual. Life Outcomes* **15**, 1–8 (2017). 28705225 10.1186/s12955-017-0715-8 PMC5513118

[4] Levasseur, M., Desrosiers, J. & Noreau, L. Is social participation associated with quality of life of older adults with physical disabilities? *Disabil. Rehabil.* **26**, 1206–1213 (2004). 15371021 10.1080/09638280412331270371

[5] Levasseur, M. *et al.* Scoping study of definitions of social participation: Update and co-construction of an interdisciplinary consensual definition. *Age Ageing* **51**, 1–13 (2022). 10.1093/ageing/afab215 PMC9383398 35134843

[6] He, T., Huang, C., Li, M., Zhou, Y. & Li, S. Social participation of the elderly in China: The roles of conventional media, digital access and social media engagement. *Telemat. Inform.* **48**, 101347 (2020).

[7] Yeung, J. W. K., Zhang, Z. & Kim, T. Y. Volunteering and health benefits in general adults: Cumulative effects and forms. *BMC Public Health* **18**, 1–8 (2017). 28693551 10.1186/s12889-017-4561-8 PMC5504679

[8] Menassa, M. *et al.* Concepts and definitions of healthy ageing: A systematic review and synthesis of theoretical models. *eClinicalMedicine* **56**, 101821 (2023). 36684393 10.1016/j.eclinm.2022.101821 PMC9852292

[9] Rowe, J. W. & Kahn, R. L. Successful ageing. *Gerontologist* **37**, 433–440 (1997). 9279031 10.1093/geront/37.4.433

[10] Urtamo, A., Jyväkorpi, S. K. & Strandberg, T. E. Definitions of successful ageing: A brief review of a multidimensional concept. *Acta Biomed.* **90**, 359–363 (2019). 31125022 10.23750/abm.v90i2.8376 PMC6776218

[11] Danquah, E. & Asiamah, N. Associations between physical work environment, workplace support for health, and presenteeism: A COVID-19 context. *Int. Arch. Occup. Environ. Health* **95**, 1807–1816 (2022). 35570224 10.1007/s00420-022-01877-1 PMC9108018

[12] Mao, L. & Normand, C. The effect of volunteering on employment: Evidence from the Irish Longitudinal Study on ageing (TILDA). *J. Econ. Ageing* **21**, 100350 (2022).

[13] Asiamah, N., Kouveliotis, K., Eduafo, R. & Borkey, R. The influence of community-level built environment factors on active social network size in older adults: Social activity as a moderator. *Int. Q. Community Health Educ.* **41**, 77–87 (2020). 32741318 10.1177/0272684X20915379

[14] Van Der Cammen, T. J. M., Albayrak, A., VoûTe, E. & Molenbroek, J. F. M. New horizons in design for autonomous ageing. *Age Ageing* **46**, 11–17 (2017). 28181640 10.1093/ageing/afw181

[15] Rudnicka, E. *et al.* The World Health Organization (WHO) approach to healthy ageing. *Maturitas* **139**, 6–11 (2020). 32747042 10.1016/j.maturitas.2020.05.018 PMC7250103

[16] Brañas, F. *et al.* Frailty and physical function in older HIV-infected adults. *Age Ageing* **46**, 522–526 (2017). 28203694 10.1093/ageing/afx013

[17] Muszyńska, M. M. & Rau, R. The old-age healthy dependency ratio in Europe. *J. Popul. Ageing* **5**, 151–162 (2012). 22924086 10.1007/s12062-012-9068-6 PMC3412045

[18] Zacher, H. Successful aging at work. *Work Aging Retire.* **1**, 4–25 (2015).

[19] Sghaier, S., Asiamah, N., Danquah, E., Opuni, F. F. & Hatsu, S. Information technology ability mediates the association between older adults’ subjective age and social activity: A STROBE-compliant cross-sectional analysis. *Arch. Gerontol. Geriatr.* **103**, 104790 (2022). 35987033 10.1016/j.archger.2022.104790

[20] Yu, T. K. & Chao, C. M. Assessing older adults’ information technology ability: The development of a multiple item scale. *Int. J. Hum. Comput. Interact.* **30**, 435–445 (2014).

[21] Siddiq, H., Teklehaimanot, S. & Guzman, A. Social isolation, social media use, and poor mental health among older adults, California Health Interview Survey 2019–2020. *Soc. Psychiatry Psychiatr. Epidemiol.* **59**, 969–977 (2024). 37728756 10.1007/s00127-023-02549-2 PMC11116239

[22] Holt-Lunstad, J. Fostering social connection in the workplace. *Am. J. Health Promot.* **32**, 1307–1312 (2018). 29972035 10.1177/0890117118776735a

[23] Berraies, S., Lajili, R. & Chtioui, R. Social capital, employees’ well-being and knowledge sharing: Does enterprise social networks use matter? Case of Tunisian knowledge-intensive firms. *J. Intellect. Cap.* **21**, 1153–1183 (2020).

[24] Matias, J. *et al.* The use of multi-national web surveys for comparative analysis: Lessons from the European Web survey on drugs. *Int. J. Drug Policy* **73**, 235–244 (2019). 30979658 10.1016/j.drugpo.2019.03.014

[25] Gharawi, M. A., Pardo, T. A. & Guerrero, S. Issues and strategies for conducting cross-national e-Government comparative research. In *ACM Int. Conf. Proceeding Ser.* 163–170. 10.1145/1693042.1693076 (2009).

[26] Casas, F. & González-Carrasco, M. *Analysing Comparability of Four Multi-Item Well-Being Psychometric Scales Among 35 Countries Using Children’s Worlds 3rd Wave 10 and 12-Year-Olds Samples. Child Indicators Research* Vol. 14 (Springer, 2021).

[27] Cerin, E. *et al.* Development and validation of the neighborhood environment walkability scale for youth across six continents. *Int. J. Behav. Nutr. Phys. Act.* **16**, 1–16 (2019). 31796075 10.1186/s12966-019-0890-6 PMC6892154

[28] Sharma, A. *et al.* A consensus-based checklist for reporting of survey studies (CROSS). *J. Gen. Intern. Med.* **36**, 3179–3187 (2021). 33886027 10.1007/s11606-021-06737-1 PMC8481359

[29] Schmidt, S. A. J., Lo, S. & Hollestein, L. M. Research techniques made simple: Sample size estimation and power calculation. *J. Investig. Dermatol.* **138**, 1678–1682 (2018). 30032783 10.1016/j.jid.2018.06.165

[30] Asiamah, N., Mensah, H. K., Fosu Oteng-Abayie, E. & Kofi Mensah, H. Do larger samples really lead to more precise estimates? A simulation study. *Am. J. Educ. Res.* **5**, 9–17 (2017).

[31] Ferguson, L. External validity, generalizability, and knowledge utilization. *J. Nurs. Scholarsh.* **36**, 16–22 (2004). 15098414 10.1111/j.1547-5069.2004.04006.x

[32] Kyriazos, T. A. Applied psychometrics: Sample size and sample power considerations in factor analysis (EFA, CFA) and SEM in general. *Psychology* **09**, 2207–2230 (2018).

[33] Jakobsen, M. & Jensen, R. Common method bias in public management studies. *Int. Public Manag. J.* **18**, 3–30 (2015).

[34] Kock, F., Berbekova, A. & Assaf, A. G. Understanding and managing the threat of common method bias: Detection, prevention and control. *Tour. Manag.* **86**, 104330 (2021).

[35] Newman, D. A. Missing data: Five practical guidelines. *Organ. Res. Methods* **17**, 372–411 (2014).

[36] Vatcheva, P. K. & Lee, M. Multicollinearity in regression analyses conducted in epidemiologic studies. *Epidemiol. Open Access* **06**, 1–20 (2016). 10.4172/2161-1165.1000227 PMC4888898 27274911

[37] Kim, K., Buckley, T., Burnette, D., Kim, S. & Cho, S. Measurement indicators of age-friendly communities: Findings from the AARP age-friendly community survey. *Gerontologist* **62**, e17–e27 (2022). 33909074 10.1093/geront/gnab055 PMC8759505

[38] Kava, C. M., Passey, D., Harris, J. R., Chan, K. C. G. & Peggy, A. The workplace support for health scale: Reliability and validity of a brief scale to measure employee perceptions of wellness. *Am. J. Health Promot.* **35**, 179–185 (2021). 32808553 10.1177/0890117120949807 PMC7870498

[39] Perera, B. P. R., Jayasuriya, R., Caldera, A. & Wickremasinghe, A. R. Assessing mental well-being in a Sinhala speaking Sri Lankan population: Validation of the WHO-5 well-being index. *Health Qual. Life Outcomes* **18**, 1–9 (2020). 32912245 10.1186/s12955-020-01532-8 PMC7488505

[40] Ferreira, A. C. L., Pereira, D. S., da Silva, S. L. A., Carvalho, G. A. & Pereira, L. S. M. Validity and reliability of the short form brief pain inventory in older adults with nociceptive, neuropathic and nociplastic pain. *Geriatr. Nurs.* **52**, 16–23 (2023). 37192570 10.1016/j.gerinurse.2023.04.011

[41] Asiamah, N., Adu-Gyamfi, K., Frimpong, F. K. S. & Avor, W. M. K. Development of a scale measuring nurses’ physical activity counseling in a primary care facility: Implications for healthcare quality. *Hosp. Top.* **99**, 119–129 (2021). 33459201 10.1080/00185868.2020.1871575

[42] Kyriazos, T. A. Applied psychometrics: The 3-faced construct validation method, a routine for evaluating a factor structure. *Psychology* **09**, 2044–2072 (2018).

[43] Bempong, A. E. & Asiamah, N. Neighbourhood walkability as a moderator of the associations between older Ghanaians’ social activity, and the frequency of walking for transportation: A cross-sectional study with sensitivity analyses. *Arch. Gerontol. Geriatr.* **100**, 104660 (2022). 35182991 10.1016/j.archger.2022.104660

[44] Sims, T., Reed, A. E. & Carr, D. C. Information and communication technology use is related to higher well-being among the oldest-old. *J. Gerontol. Ser. B Psychol. Sci. Soc. Sci.* **72**, 761–770 (2017). 27702839 10.1093/geronb/gbw130

[45] Pruchno, R. Technology and aging: An evolving partnership. *Gerontologist* **59**, 1–5 (2019). 30629258 10.1093/geront/gny153

[46] Kim, J., Lee, H. Y., Candace Christensen, M. & Merighi, J. R. Technology access and use, and their associations with social engagement among older adults: Do women and men differ? *J. Gerontol. Ser. B Psychol. Sci. Soc. Sci.* **72**, 836–845 (2017). 28073816 10.1093/geronb/gbw123

[47] Guzman, A. A., Brecht, M. L., Doering, L. V., Macey, P. M. & Mentes, J. C. Social media use and depression in older adults: A systematic review. *Res. Gerontol. Nurs.* **16**, 97–104 (2023). 36944173 10.3928/19404921-20230220-05

[48] Yao, J. & Cao, X. The balancing mechanism of social networking overuse and rational usage. *Comput. Hum. Behav.* **75**, 415–422 (2017).

[49] Asiamah, N., Bateman, A., Hjorth, P., Khan, H. T. A. & Danquah, E. Socially active neighborhoods: Construct operationalization for aging in place, health promotion and psychometric testing. *Health Promot. Int.* **38**, 1–10 (2023). 10.1093/heapro/daac191 PMC9933838 36795097

[50] Asiamah, N. Social engagement and physical activity: Commentary on why the activity and disengagement theories of ageing may both be valid. *Cogent Med.* **4**, 1289664 (2017).

[51] Torkzadeh, G. & Van Dyke, T. P. *Effects of Training on Internet Self-Efficacy and Computer User Attitudes*. www.elsevier.com/locate/comphumbeh.

[52] Vaportzis, E., Clausen, M. G. & Gow, A. J. Older adults perceptions of technology and barriers to interacting with tablet computers: A focus group study. *Front. Psychol.* **8**, 1–11 (2017). 29071004 10.3389/fpsyg.2017.01687 PMC5649151

