# Supplementary material for: Association of information technology ability, workplace social engagement, and successful ageing: validation of a short measure with three African samples
Source: Sci Rep. 2024 Aug 13;14:18787. doi: 10.1038/s41598-024-69133-9 (PMC11322284; doi:10.1038/s41598-024-69133-9)
Supplement: Supplementary file 1 — Supplementary Information 1. [file 41598_2024_69133_MOESM1_ESM.doc]

**Appendix A**

**Appendix A1. Items for measuring workplace social engagement: Original and adapted**

| No. | Original item | Adapted item |
| --- | --- | --- |
| 1 | I interacted with friends in my community | I interacted with colleagues or friends at work |
| 2 | I played games (e.g., Ludo, Oware) with others | I played a game or performed a sporting activity (e.g., walking) with colleagues or friends |
| 3 | I went to an organized event (e.g. cinema, sports, drama, etc.) | I went to a performance-based event (e.g., seminar, conference, training) organized at work or by my employer |
| 4 | I provided help to a family, friend, or acquaintance | Provide help to friends, colleagues, or workmates |
| 5 | I catered for the sick or a disabled person | I catered for a colleague or friend who was disadvantaged (e.g., disabled or had a special need) at work |
| 6 | I made new friends | I made new friends at work |
| 7 | Participate in community-related events (e.g., cleaning) | I participated in non-work-related or recreational events with a friend or colleague |
| 8 | Participate in charity or voluntary work | I participated in voluntary work at work to contribute to the overall success of my organization |

Descriptive anchors to the adapted scale: 1 – not at all, 2 – less frequently, 3 – frequently, and 4 – very frequently

**Appendix A2. The Successful ageing Index or Scale**

| No | Item | 1 | 2 | 3 | 4 | 5 |
| --- | --- | --- | --- | --- | --- | --- |
| Illness avoidance | | | | | | |
| 1 | Bodily pain did not affect my performance of work or other essential tasks. |  |  |  |  |  |
| 2 | I did not use medication or therapy. |  |  |  |  |  |
| 3 | I was healthy enough to move around freely. |  |  |  |  |  |
| 4 | I had good health overall. |  |  |  |  |  |
| Functioning | | | | | | |
| 5 | I had enough energy for daily life. |  |  |  |  |  |
| 6 | I have been sleeping well. |  |  |  |  |  |
| 7 | When I tried to recall familiar names or words, it was not difficult for me to do so. |  |  |  |  |  |
| 8 | I could perform two or more tasks simultaneously, for example, watch TV while discussing something else with another person. |  |  |  |  |  |
| 9 | My body and mind were strong enough to enable me to live independently without having others to take care of me. |  |  |  |  |  |
|  | Engagement with life |  |  |  |  |  |
| 10 | I provided concern and support to enrich the lives of nuclear family members (e.g., husband or wife). |  |  |  |  |  |
| 11 | I provided concern and support to enrich the lives of family extended family members (e.g., niece or uncle). |  |  |  |  |  |
| 12 | I provided concern and support to enrich the lives of my neighbours. |  |  |  |  |  |
| 13 | I provided concern and support to enrich the lives of friends, colleagues, or workmates. |  |  |  |  |  |
| 14 | Overall, I was concerned about and supportive to people around me to enrich their lives. |  |  |  |  |  |
| 15 | I make financial or productive contribution to my family. |  |  |  |  |  |
| 16 | I make financial or productive contribution to my career and work. |  |  |  |  |  |
| 17 | I make financial or productive contribution to my community or non-profit making organizations. |  |  |  |  |  |
| 18 | Overall, I contributed to society as a whole (including contributions made to your family, your career and work, and the community). |  |  |  |  |  |

Descriptive anchors: 1 – strongly disagree, 2 – disagree, 3 – somewhat agree, and 4 – agree, and 5 – strongly agree.

**Appendix A3: Items for measuring information technology ability**

| No. | Statement | 1 | 2 | 3 | 4 | 5 |
| --- | --- | --- | --- | --- | --- | --- |
| 1 | In general, I routinely use the Internet to obtain good information. |  |  |  |  |  |
| 2 | I commonly use the Internet to quickly retrieve useful information. |  |  |  |  |  |
| 3 | I would use the Internet to quickly mail attached files to friends. |  |  |  |  |  |
| 4 | I use the Internet because it is easy to get information that relates to my needs. |  |  |  |  |  |
| 5 | I would use the Internet because getting required information is inexpensive. |  |  |  |  |  |
| 6 | I could share ideas and thoughts on specific topics through package software. |  |  |  |  |  |
| 7 | I could apply word-processing software in document editing. |  |  |  |  |  |
| 8 | I could use package software to publish documentation on the Internet. |  |  |  |  |  |
| 9 | I could describe the package software functions. |  |  |  |  |  |
| 10 | I like to experiment with new technologies. |  |  |  |  |  |
| 11 | In general, I am hesitant to try out new technologies. |  |  |  |  |  |
| 12 | If I heard about new technologies, I would look for ways to experiment with them. |  |  |  |  |  |
| 13 | Among my peers, I am usually the first to try out new technologies. |  |  |  |  |  |

Descriptive anchors: 1 – strongly disagree, 2 – disagree, 3 – somewhat agree, 4 – agree, and 5 – strongly agree; Domains: items 1-5 measure internet use assessment; items 6-9 measure packaged software use assessment, and items 10-13 measure personal innovativeness attitude.
