# Supplementary material for: Association of information technology ability, workplace social engagement, and successful ageing: validation of a short measure with three African samples
Source: Sci Rep. 2024 Aug 13;14:18787. doi: 10.1038/s41598-024-69133-9 (PMC11322284; doi:10.1038/s41598-024-69133-9)
Supplement: Supplementary file 2 — Supplementary Information 2. [file 41598_2024_69133_MOESM2_ESM.doc]

Appendix B

Appendix B1. Steps to taken to test five necessary assumptions for HLR analysis

| # | Assumption | Step | Result | Decision |
| --- | --- | --- | --- | --- |
| 1 | Normality of the data associated with the dependent variable | We computed the Mahalanobis values through the HLR in which successful ageing was the dependent variable | The significance values associated with the Mahalanobis values met the condition p<0.001; thus normality was confirmed (Bempong & Asiamah, 2022; Sghaier et al., 2022) | We proceeded with the parametric analysis since the condition was met. |
| 2 | Linearity | We plotted standardized residuals against standardized predicted values of the dependent variable in the above HLR analysis. We observed the linearity of the lines of best fit. | The graph shows a linear pattern as recommended (Sghaier et al., 2022) | Assumption or condition met for HLR analysis |
| 3 | Independence of errors | Durbin Watson statistics were generated for all the HLR models fitted. | Durbin-Watson statistic was approximately 2 for each multiple regression model as recommended (Bempong & Asiamah, 2022) | The assumption was met for HLR analysis |
| 4 | Multi-collinearity | Tolerance values were computed through the above HLR analysis | The tolerance values are >0.2 as recommended (Bempong & Asiamah, 2022) | The assumption was met for HLR analyses |
| 5 | Homogeneity of variances | We plotted standardized residuals against standardized predicted values of the dependent variable in all HLR models | The graphs produced a satisfactory pattern as recommended (Bempong & Asiamah, 2022) | The assumption was met for HLR analyses |

**Note**: HLR – hierarchical linear regression

Appendix B2. Procedures followed in the first sensitivity analysis for confounding variables

| Stage | # | Action |
| --- | --- | --- |
| 1 | 1 | Fit a simple linear regression model to evaluate the association between WSE and successful ageing |
|  | 2 | Note the standardised regression weight from step 1 |
|  | 3 | Fit a multiple linear regression model that treats all the measured confounding variables as predictors of WSE |
|  | 4 | Identify from step 3 potential confounders that have a p-value ≥0.25 |
|  | 5 | Predictors from step 4 that produced a p≥0.25 should be removed from the analysis and the others kept for the next stage of the analysis |
| 2 | 6 | Control for each remaining confounding variable by infusing it in the model at step 1 as a co-predictor |
|  | 7 | Calculate the per cent change between the standardised regression weight at step 1 and the new weight resulting from step 6 |
|  | 8 | All potential confounders that produce a change of 10% or more should be incorporated into the final analysis as the ultimate confounders |

Note: WSE – workplace social engagement
