# Supplementary material for: Association of information technology ability, workplace social engagement, and successful ageing: validation of a short measure with three African samples
Source: Sci Rep. 2024 Aug 13;14:18787. doi: 10.1038/s41598-024-69133-9 (PMC11322284; doi:10.1038/s41598-024-69133-9)
Supplement: Supplementary file 3 — Supplementary Information 3. [file 41598_2024_69133_MOESM3_ESM.doc]

Appendix C.

Appendix C1. Descriptive statistics and reliability indicators

| Variable | Wave 1 (Ghana, n = 161) | | | Wave 2 (Consolidated, n = 1184) | | |
| --- | --- | --- | --- | --- | --- | --- |
|  | Mean | SD | Factor loading | Mean | SD | Factor loading |
| 1. I interacted with colleagues or friends at work | 2.94 | 1.10 | 0.75 | 3.20 | 0.92 | 0.61 |
| 2. I played a game or performed a sporting activity (e.g., walking) with colleagues or friends | 2.53 | 1.16 | 0.77 | 2.60 | 1.01 | 0.71 |
| 3. I went to a performance-based event (e.g., seminar, conference, training) organized at work | 2.77 | 1.06 | 0.71 | 2.61 | 0.98 | 0.62 |
| 4. Provide help to friends, colleagues, or workmates | 2.89 | 0.95 | 0.84 | 2.98 | 0.90 | 0.67 |
| 5. I catered for a colleague or friend who was disadvantaged (e.g., disabled or had a special need) at work | 2.59 | 1.00 | 0.71 | 2.54 | 0.92 | 0.62 |
| 6. I made new friends at work | 2.62 | 1.08 | 0.73 | 2.78 | 1.04 | 0.67 |
| 7. I participated in non-work-related or recreational events with a friend or colleague | 2.60 | 1.08 | 0.81 | 2.63 | 0.96 | 0.66 |
| 8. I participated in voluntary work at work to contribute to the overall success of my organization | 2.46 | 1.02 | 0.77 | 2.89 | 0.99 | 0.62 |

Wave 1: total variance – 58.12; Cronbach’s alpha – 0.898; Wave 2: total variance – 41.92%; Cronbach’s alpha – 0.801

Appendix C2. Correlation matrix of items of WSE at wave 1 (n = 161) and wave 2 (n = 1184)

| Variables | 1 | 2 | 3 | 4 | 5 | 6 | 7 | 8 |
| --- | --- | --- | --- | --- | --- | --- | --- | --- |
| Wave 1 (Ghana, 161) | | | | | | | | |
| 1. I interacted with colleagues or friends at work | 1 |  |  |  |  |  |  |  |
| 2. I played a game or performed a sporting activity (e.g., walking) with colleagues or friends | 0.602** | 1 |  |  |  |  |  |  |
| 3. I went to a performance-based event (e.g., seminar, conference, training) organized at work | 0.434** | 0.479** | 1 |  |  |  |  |  |
| 4. Provide help to friends, colleagues, or workmates | 0.658** | 0.57** | 0.458** | 1 |  |  |  |  |
| 5. I catered for a colleague or friend who was disadvantaged (e.g., disabled or had a special need) at work | 0.551** | 0.469** | 0.43** | 0.579** | 1 |  |  |  |
| 6. I made new friends at work | 0.405** | 0.495** | 0.546** | 0.515** | 0.355** | 1 |  |  |
| 7. I participated in non-work-related or recreational events with a friend or colleague | 0.471** | 0.559** | 0.534** | 0.693** | 0.519** | 0.539** | 1 |  |
| 8. I participated in voluntary work at work to contribute to the overall success of my organization | 0.46** | 0.496** | 0.497** | 0.604** | 0.47** | 0.598** | 0.562** | 1 |
| Wave 2 (Consolidated, n = 1184) | | | | | | | | |
| 1. I interacted with colleagues or friends at work | 1 |  |  |  |  |  |  |  |
| 2. I played a game or performed a sporting activity (e.g., walking) with colleagues or friends | .34** | 1 |  |  |  |  |  |  |
| 3. I went to a performance-based event (e.g., seminar, conference, training) organized at work | .26** | .38** | 1 |  |  |  |  |  |
| 4. Provide help to friends, colleagues, or workmates | .38** | .42** | .18** | 1 |  |  |  |  |
| 5. I catered for a colleague or friend who was disadvantaged (e.g., disabled or had a special need) at work | .30** | .33** | .45** | .22** | 1 |  |  |  |
| 6. I made new friends at work | .25** | .43** | .33** | .49** | .26** | 1 |  |  |
| 7. I participated in non-work-related or recreational events with a friend or colleague | .30** | .38** | .40** | .32** | .47** | .27** | 1 |  |
| 8. I participated in voluntary work at work to contribute to the overall success of my organization | .36** | .33** | .23** | .408** | .22** | .41** | .29** | 1 |

**p<0.001

Appendix C3. The correlation of WSE with engagement with life, self-reported health, and physical functional ability at waves 1 and 2

**p<0.001

| Variable | 1 | 2 | 3 |
| --- | --- | --- | --- |
| Wave 1 (Ghana, n = 161) | | | |
| 1. Workplace social engagement | 1 |  |  |
| 2. Self-reported Health | .268** | 1 |  |
| 3. Engagement with life | .514** | .222** | 1 |
| Wave 2 (n = 1184) | | | |
| 1. Workplace social engagement | 1 |  |  |
| 2. Self-reported Health | .145** | 1 |  |
| 3. Engagement with life | .439** | .238** | 1 |
